# On Tracheotomy in Croup

**Published:** 1840-01-04

**Authors:** William P. Johnston

**Affiliations:** Georgia


MEDICAL EXAMINER.

DEVOTED TO MEDICINE, SURGERY, AND THE COLLATERAL SCIENCES.

No. 1.] PHILADELPHIA, SATURDAY, JANUARY 4, 1839. [Vol. III.

### ON TRACHEOTOMY IN CROUP.

By William P. Johnston, M. D., of Georgia.

To the Editors of the Medical Examiner.

Gentlemen,—In the accompanying essay on tracheotomy which I have the honour to send you, will be found many operations cited without a reference being given—these cases, composing, indeed, the majority, are not on record: for a knowlege of them I am chiefly indebted to the politeness of M. Trousseau.

Among the ancient medical writers we find no well authenticated case of cure following the operation of tracheotomy in croup, although it appears to have been occasionally resorted to for centuries. In the latter part of the last century, and in the beginning of the present one, this operation was more frequently repeated both in Europe and in America,—but with so little success, that it continued to be discountenanced by the majority of the profession, and regarded with horror by many. Within the last few years, however, the attention of physicians has been awakened to the importance of this ultimate resource in the treatment of croup; and medical opinion, in France at least, is undergoing a complete revolution. The honour of having revived the operation of tracheotomy is justly due to M. Bretonneau of Tours, to whom we are also indebted for one of the most able treatises on this disease written in the French language. Having carefully studied the pathological characters of the diphtheritis of children, and then closely observed the effect of the various methods of treatment generally employed, M. Bretonneau soon became convinced that topical remedies, "when they could be applied over the whole extent of the disease," were the measures chiefly to be depended upon; and hence he concluded that when the disease had reached the larynx, reasonable hopes of success might still be entertained, could he but prolong the lives of his patients by an operation, while he availed himself of the artificial opening thus made to continue his local applications to better advantage. In order to test the value of his theoretical ideas, M. B. commenced performing tracheotomy in 1818; but it was not until 1825 that he succeeded, in his third case, in effecting a complete cure. It may be well to remark here, that this operation had once already been successfully performed in London, by John André, in 1782. This is the first authenticated cure which we can find on record.

In 1826, M. Trousseau, now professor of materia medica and therapeutics in the Medical School of Paris, warmly espoused the propriety of tracheotomy, and endeavoured, by modifying the method of operating, of introducing the canula, &c. &c., to avoid as much as possible the accidents which were so generally dreaded, while, at the same time, he hoped, by systematizing the subsequent treatment in the minutest particulars, to increase the chances of success. In 1833 M. T. published in the Journal des Connaiss. Med. Chir. his first case of cure which he had obtained two years previously: since this period he has repeated tracheotomy very frequently, and we shall see presently with what success. In 1834, Professor Gerdy's first case of cure was published in the Archives of Medicine. Since this period, tracheotomy has been performed in croup by most of the leading men in Paris, who unite almost unanimously in declaring themselves partisans of the operation. Thus Guersent, (Dict. Med., 2d ed. t. ix,) makes use of the following words in reference to it:—"Every time that I have seen this operation performed, even without success, I have remarked a sensible amelioration, at least during several hours, and sometimes during several days." At the meeting of the Academy of Medicine on the 21st May last, M. Bricheteau, in a report made upon the subject of which we treat, remarked, that in his treatise on croup, published in 1826, he had censured the opening of the trachea, but acknowledged that the numerous recent successful cases of tracheotomy had convinced him of his error in this respect, and that he would now recommend, on the contrary, not deferring it too long. At the same meeting, Velpeau, Gerdy, Blandin, and Amussat, expressed an opinion similar to that of Guersent, which we have quoted above, namely, that they approved of the operation,—that it appeared to prolong life, even when it ultimately failed,—and that they should continue to perform it, should cases offer themselves. It will be needless for us to multiply names; it is sufficient to say, that in Paris, the profession at large entertain the same view as to the propriety of tracheotomy: there are doubtless exceptions; but among those who have expressed themselves upon the subject within the last two or three years, we know not one who is not willing to admit its utility, at least in certain cases.

Having thus given, in a summary manner, the progress of the operation in France, and the prevalent opinion in Paris at the present moment as to its real utility; we shall now endeavour to put in a tabular form the names of the different individuals who have performed tracheotomy, with the exact number, as far as possible, of each one's successful cases.

| No. | Names. | Residence. | No. of operations. | No. of cures. | References and Remarks. |
|---|---|---|---|---|---|
| 1 | Trousseau. | Paris. | 92 | 23 | See table containing 30 operations in the Journal des Conn. Med. Chir., Sept., 1834; remaining cases not published. |
| 2 | Bretonneau. | Tours. | 38 | 11 | Five cases only, published in his treatise on croup; 17 noticed by Guersent. |
| 3 | Gerdy. | Paris. | 6 | 4 | One published in the Archives Gen. de Med., 1835, t. v. |
| 4 | Velpeau. | Do. | 7 | 1 | Séance de l'Academie de Med. du 21 Mai. The seventh and successful operation was performed about three months since. |
| 5 | Gendron, (de Vendome.) | | 4 | 1 | Acad. Royale de Med., 1838, t. iii.; Archives de Med., Nov., 1839; Journ. des Conn. Med. Chir., Nov., 1835. |
| 6 | Nobis. | | 2 | 1 | |
| 7 | MM. Meunier and Antoine. | Chateau d'Eau. | 1 | 1 | Recently performed. (7–9) |
| 8 | Nelaton. | Paris. | 1 | 1 | |
| 9 | Demaux. | Interne à l'hôpital des Enfans Mal. | 1 | 1 | |
| 10 | Dr. Mott. | New York. | 1 | 1 | |
| 11 | Blandin. | Paris. | 5 | 0 | Séance de l'Acad. de Med., 21 Mai, 1839. (11–12) |
| 12 | Amussat. | Do. | 5 | 0 | |
| 13 | Guersent, (fils.) | Do. | 11 | 0 | One patient lived to the eighth day; a second to the fourteenth: in the latter the wound was nearly healed. |
| 14 | | A l'hôpital des Enfans. | 15 | 0 | Cited by Baudelocque at the sitting of the Academie of Medicine in May last. |
| 15 | Boneface. | Paris. | ? | 1 | |
| 16 | Scoutetten. | Metz. | ? | 1 | Coquerel, These, No. 185, Paris, 1834. |
| 17 | Senn. | Geneve. | ? | 1 | |
| 18 | Bulliard. | | ? | 1 | Archives de Med., 7 aunée, t. xix. |
| 19 | Crozat. | | ? | 1 | Précis Med. d'Indre et Loire, quoted by Velpeau. |
| 20 | André. | London. | ? | 1 | Reported in Borsieri. |
| 21 | Chevalier. | Do. | ? | 1 | See last edition of the Dictionary of Samuel Cooper. (21–22) |
| 22 | S. Cooper, &c. | Do. | ? | 1 | |
| 23 | Bazin. | Paris. | ? | 1 | Journal de l'Esculape? |
| 24 | Gendron. | Du Chateau du Loir | ? | | Archives, t. v., 3 serie, p. 363. Death eleven days after operation, from cause totally foreign to the disease and to the operation. |
| 25 | Mazier. | | ? | | Annal. de la Med. Phys., Dec., 1828. Death from escape of canula; the respiration had become very easy. |
| 26 | Sanson, (ainé.) | Paris. | ? | ? | Said by Trousseau to have operated. (Dict. de Med., 2d ed., t. ix., *Croup.*) |
| | | | 201? | 54 | |

If we examine the table which we have here given for the purpose of ascertaining the relation which exists between the number of cases and the whole number of operations, we shall find that tracheotomy is known to have been performed a little more than two hundred times; and to have succeeded fifty-four times; which gives us an average of one cure in less than four operations. But this is not the true proportion, for in several instances, in the above table, the whole number of operations are not mentioned. We will suppose, for an instant, that in each of the twelve cases, where this deficiency exists, the operation was repeated five times; this will give us two hundred and forty-nine operations and fifty-four cures, or one successful case in four and eleven-eighteenths, a proportion which still speaks highly in favour of tracheotomy. Thus far we have examined our cases in the most unfavourable light; for if we confine our attention to those only where we find the exact number of operations given we shall have a greater average of cures; thus, by taking only the first fourteen in the table we shall have forty-five cures for one hundred and eighty-nine operations, that is, one for every four and one-fifth. But this is still not absolutely correct, for we find that M. Trousseau mentions, in the Journal des Conn. Med. Chir., Sept. 1834, that three times the operation had been performed by him when the patients had already expired, and with a view of recalling life, and that three other patients, who gave the most flattering hopes of complete success, died in a few minutes through the inexperience of the assistants, who could not replace the canulas which had escaped during the paroxysms of cough. These six cases of M. T. ought necessarily to be omitted in making a fair calculation; substracting them therefore from the one hundred and eighty-nine, we shall have left one hundred and eighty-three operations and forty-five cures, equal to one in four and one-fifteenth.

We cannot see what further argument is necessary to prove the utility of tracheotomy. Facts cannot mislead, and the large mass of them which we have succeeded in collecting must, we think, bring conviction to the mind of every one. We have only to bear in mind one point, namely, that tracheotomy has been the means of saving the lives of nearly one-fourth of the patients, otherwise doomed to an almost certain death. This, indeed, is literally true, for we have only to read the cases which have been published upon this subject to feel assured how vain would have been any other hope. It is from having seen this operation successfully performed in several cases, and from a careful perusal of all that we have found for and against it, that we ourselves have been forced to look upon tracheotomy as a last resource, but one never to be neglected. We repeat again, one-fourth of the children operated upon have been saved by it; and we may add, that if the proportion had been but half as great, we should still say, operate, nothing is to be lost, much may be gained.

A question naturally suggests itself, in this place, relative to the want of success which has followed the operations of Guersent, (fils) Amussat, Blandin, and those performed at the children's hospital. M. Velpeau (Med. Operat. 2d edit. Tracheotomy,) thinks with M. Maingault, "that the operation is generally performed too late, and that the consecutive treatment claims too many precautions for it to succeed in the hands of all so well as with MM. Bretonneau and Trousseau." A most scrupulous attention to the patient after tracheotomy is indeed a *sine qua non* to success, and to the want of this alone we may reasonably attribute many failures, especially in the children's hospital.

There are but few circumstances which may be regarded as positive contra-indications to the operation; of these the most important, doubtless, are an extensive double pneumonia, and the existence of an advanced tuberculous affection of the lungs; in either case death must be certain, as M. Guersent has well remarked, and it would, therefore, be needless to have recourse to tracheotomy. If there be pneumonia of one side only, M. Guersent still recommends operating, though there will be but little chance of success.

M. Rochoux (Gazette des Hopitaux du 23 Mai) and M. Gendron (Jour. des Conn. Med. Chir. Nov. 1835) both regard tracheotomy as useless in those cases where the disease has commenced in the ramifications of the bronchiæ, and spread from thence to the trachea and larynx; others regard tracheotomy as contra-indicated the moment the false membranes have reached the bronchiæ; supposing, as is generally the case, that the disease has commenced from some point above the larynx. The same answer may be made to both of these opinions, namely, that we cannot distinguish in every case, as M. Gendron himself allows, whether the asphyxia proceeds from an obstacle in the larynx, trachea, or bronchiæ; and, besides, could we make always a rigorous diagnosis, in this respect, the existence of false membranes in the bronchiæ would still be no absolute contra-indication, though the chances of success would be diminished, especially if they should be very adherent; our opinion in this respect is founded upon the fact that M. Trousseau has himself effected three cures under similar circumstances. M. T. was kind enough to show me, a few days since, the false membrane which he had extracted from one of those patients. This membrane is of a tubular form; it exhibits the incision made into it by the bistoury through which the canula itself had been introduced; it appears to have extended from a little above the artificial opening made in the trachea to the distance of several inches; below it divides into two principal branches, corresponding to the division of the bronchiæ. The most remarkable thing in this preparation is the subdivision of the bronchial branches of the false membrane into several ramifications, in which we distinguish a second and third order. This one fact alone would be sufficient, in the absence of others, to warrant us in declaring, *that the extent of the disease alone should never be considered a contra-indication to tracheotomy.* It may not be uninteresting to mention here a pathological fact relative to this disease, founded upon the researches of MM. Hussenot and Guersent (Dict. Med., Croup.) viz. that in the majority of cases the affection does not extend beyond the trachea; thus, in one hundred and twenty autopsies the false membrane was found to have reached the bronchiæ in forty-two only, while in seventy-eight they remained confined to the larynx and trachea, a result, as M. Guersent remarks, very favourable to tracheotomy.

Too great feebleness on the part of the patient, a debilitated and depraved constitution, if not absolute contraindications, leave us at least but little to hope from the operation, especially if the disease occurs among the haunts of poverty and misery where necessary attention cannot be obtained. It does not come within the scope of our proposed plan to speak at length upon the treatment of croup, but there is a remedy in this disease often resorted to, and sometimes, perhaps, abused, which it is important to notice, as it exercises a most injurious influence in the final result after tracheotomy. We refer to the too copious abstraction of blood either by the lancet or by leeches. From an analysis of thirty cases relative to this point, (Journal des Conn. Med. Chir. Sept. 1834) M. Trousseau found that all those patients had died who had been greatly debilitated by previous loss of blood, and that they lived, upon an average, but twenty-six hours after the operations. That nearly a half of those, on the contrary, were cured, who had not had leeches applied, or who had been bled very moderately, and that those who died, lived upon an average, thirty-eight hours. M. T. concludes from this, that too much bleeding is highly unfavourable to the success of tracheotomy. Bretonneau entertains the same opinion. We had an occasion of seeing, ourselves, a few days since, an example of the unhappy influence of a too abundant loss of blood; this was in a little boy, aged two and a half years. The patient was seen in the morning by M. Trousseau, at which time he presented the well marked symptoms of croup, but his state was by no means alarming. During the day, in the absence of M. T., another physician had been called in, who ordered leeches to the neck; a few hours afterwards we visited the patient with M. T. and found him presenting the symptoms of the last period of croup, and much prostrated by the loss of blood, which was not yet arrested. The operation was immediately judged necessary, and performed without delay; the hæmorrhage was not great; it appeared to us that the patient lost at most, two or three ounces of blood; this, however, added to what he had previously lost, threw him into a state of the greatest prostration; his pulse became scarcely sensible, and finally, such protracted syncopy came on the moment the canula was introduced into the trachea, that it was with great difficulty that he could be roused. Respiration became established in a few minutes, but the patient remained feeble and ultimately died.

Bleeding is no doubt advantageous when there is much general reaction at the commencement of the disease; hence, we find it recommended by Guersent and others in such cases, but we may say in general terms, that all those who look to tracheotomy as an ultimate resource, use this method of depletion very frugally, for the reasons which we have given.

We are far from disputing the utility which Dr. Meigs, (Medical Examiner, vol. i, p. 414) and others may have obtained from bleeding "usque ad deliquium;" our object is simply to show how unfavourable this is to the operation. It is a question, indeed, still to be determined, whether large bleedings ever shorten the natural course of the disease; the majority, we know, profess the affirmative, but it must not be forgotten that there are good observers who support the negative, among whom we may mention Bretonneau, Guersent and Gendron.

Our own opinion is that bleeding, except under the circumstances which we have mentioned above, should be avoided, and that we should rely chiefly upon cauterization, which may be carried as far as the glottis. The facts published by Bretonneau and others since, are entirely in favour of this method of treatment, which has the double advantage of often arresting the disease and never weakening the patient.

Supposing us to have decided upon the propriety of the operation, we will now examine the question, *when should it be performed?* M. Trousseau thinks that we should resort to the operation "as soon as possible," (Dict. de Med. 2d edit. t. x., Croup) that is, as soon as the existing symptoms lead us to believe that all other resources would be hopeless. "Experience has proved to me," says M. T., "that we do not lose one patient in ten when the false membrane has not extended beyond the larynx, whilst on the other hand, we scarcely save one in ten when the bronchiæ are already covered with follicular concretions." "If we practice the operation," he adds, "when the children are about to expire, we risk—1st, finding the false membrane as extended as possible; 2dly, failing in our efforts to remedy the congestion, the inflammation and the pulmonary emphysema, accidents which are common in the last degree of asphyxia; 3dly, the operation is rendered much more difficult on account of the enormous distention of the vessels of the neck, which increases in proportion to the difficulty of the respiration.

M. Guersent says, (Dict. Med. art. Croup,) "the necessity of the operation appears to me evident as soon as croup has arrived at its third period, when the cough is rare and very dry, voice extinguished, laryngo-tracheal râle dry and metallic, the inspirations high and frequent, with violent contraction of the muscles of the neck, of the alæ nasi, and heaving of the abdomen; we must not wait until there is imminent asphyxia, and too great feebleness on the part of the patient; the younger the patient, the sooner we should operate, as the disease marches much more rapidly in them than in those who approach the age of puberty and in the adult." M. Gendron, (Journal des Conn. Med. Chir. Nov. 1835) thinks that the operation should not be performed, "until the paleness of the face, the lividity, projection of the eyes, dilation of the alæ nasi, dyspnœa and frequency of the pulse, announce an imminent asphyxia." Velpeau, Gerdy, and others, repeat in general terms, that the operation should be performed early. After an attentive examination of the facts which have come under our own observation, and those which have been published in reference to this subject, we are induced to believe that the chances of success must depend, in a great measure, upon the strength of the patients, the absence of false membrane in the bronchiæ, and the healthy state of the lungs; hence we would recommend operating as soon as there was reason to think that all other treatment would be vain, or in other words, as soon as death appeared inevitable.

There is a symptom which has not been noticed by the authors that we have quoted, but which deserves to be mentioned in this place, as it may be found of great value in warning us of impending danger: we allude to the diminution of the respiratory murmur. According to M. Barth, (Archives de Med. 3 série, 1838,) the degree of the diminution of the respiratory murmur may give us a measure of the obstacle; hence the existence more or less complete of this murmur, will indicate to us that the danger is not yet extreme, whilst its diminution, more or less marked, will forewarn us of approaching or impending suffocation. We shall have to wait for further observation before we can decide upon the real value of this physical sign in the croup of children.

*Operation.*—The method which appears to us the best, is that which we have seen executed by M. Trousseau as it differs but little from the ordinary methods contained in the works on operative surgery, we shall be as brief as possible in its description.

The patient being conveniently placed in the manner usually recommended, an assistant is ordered to maintain the head of the patient so as to prevent any movement; two other aids should be present to hold the extremities, if necessary, and to separate, when required, the lips of the wound by means of blunt hooks. The operator places himself on the right of the patient, with his instruments, water and sponges, within reach: he then traces out upon the trachea of the patient, by means of a burnt cork, the course which the bistoury is to take; this line should extend from the thyroid cartillage as far as the superior extremity of the sternum, and incline towards the left side of the trachea below, so as to avoid all risk of cutting the arteria innominata; a large fold of the skin is now pinched up perpendicularly to the axis of the body, one side of which is held by the assistant placed on the left of the patient; an incision is then made towards the trachea, intersecting the fold of the skin only. The integuments being cut, the patient usually becomes tranquil; the operation is then continued by slowly and carefully dissecting the cellular tissue and the parts beneath; if a thyroid vein of any magnitude presents itself, the greatest care should be taken to avoid cutting it; for this purpose, after separating it a little from the adjoining parts, it should be carried and retained a little to one side by means of a blunt hook. Sometimes, in spite of our precaution, the hæmorrhage from the thyroid veins is very considerable; in such cases compression by means of a sponge will generally be found sufficient to moderate the effusion of blood, and to prevent the introduction of air into these vessels. Trousseau and Velpeau in particular, regard the application of ligatures as unnecessary. As for the little arteries which are divided, we have generally observed that they ceased giving out blood in a few seconds before they were even seized. If the hæmorrhage should be found to continue, tortion would doubtless be found preferable to the ligature.

When we have reached the trachea, we should separate the cellular tissue from it, in the extent that we propose to open it, by thus doing; as M. Trousseau has remarked, we avoid the subsequent emphysema of the cellular tissue, as well as the effusion of blood into the bronchiæ, which might otherwise take place from its vessels. As soon as the hæmorrhage is sufficiently arrested, a small opening is made into the trachea by means of a sharp pointed bistoury; this should be immediately withdrawn, and a probe-pointed one introduced in its place; the incision is then prolonged in the direction of the sternum, during an effort of inspiration. The opening thus made, should be immediately dilated by means of the dilator of M. Trousseau, (a kind of dressing forceps) or other instrument, and the child placed in a sitting position in order to facilitate respiration, and the discharge of mucus, &c. through the opening. A temporary syncopy often follows the operation; as soon as this has passed off, and the respiration has become freely established, the trachea, and bronchiæ, if necessary, are carefully sponged to remove the false membrane, and the mucous coat cauterized; the canula is then introduced and secured in its place, by a piece of tape passing round the neck of the child; the edges of the wound at the inferior extremity may now be brought together by means of a small piece of sticking plaster.

The size of the canula to be employed, is a point of such infinite importance, that we cannot pass it over without a few remarks. Previous to Mr. Bretonneau, the canulas in vogue, offered a very small diameter; this celebrated physician was the first to point out the numerous inconveniences which resulted from their employment. It was found that they rarely permitted a sufficient quantity of air to pass into the lungs, even at the moment of their application, and that they became so easily obstructed by fragments of false membrane and mucus, that it was necessary to remove them at every instant. Of the truth of these objections we became convinced, while Resident Physician of the Philadelphia Hospital, in the case of an adult upon whom we performed the operation of tracheotomy in consequence of an œdema of the glottis, supervening upon a chronic affection of the larynx. On this occasion, we were obliged to have an assistant constantly by the side of the patient, in order to prevent the accumulation of mucus in the instrument, for even the presence of a small quantity was sufficient to impede the respiration. Without this precaution, our patient might have died from asphyxia, and yet the secretion from the bronchiæ was not remarkable for its quantity, nor for its plasticity; it is evident that the difficulty arose from the canula not being of sufficient caliber. Bretonneau's method was to employ a large canula, so as to permit free ingress to the air, and to obviate the inconvenience of the presence of a small quantity of mucus or false membrane.

More recently, we find Trousseau and Velpeau insisting upon this point as of the first necessity. The size recommended by M. Trousseau is, for an infant at the breast, a diameter of two and a half lines at the smallest orifice; for an adult male, a diameter of six lines; between these two extremes, there ought to be at least two or three other sizes; these should be employed according to the age and physical development of the patient.

*After treatment.*—The essential points here to be considered are—1. Sponging, in order to remove the false membranes, &c. 2. Injections of tepid water to dilute the mucus, and facilitate expectoration. 3. Cauterization of the mucous membrane. 4. Diet. The directions given by M. Trousseau upon these subjects, (Dict. Med. &c.) are so full that we cannot do better than to translate them.

1. *Sponging.* "This is performed by means of a small piece of sponge placed at the extremity of a round and supple piece of whalebone, six or eight inches long; this is to be introduced through the artificial opening in the trachea, and carried rapidly to the distance of three, four, five, or even six inches, causing it to execute a movement of rotation; it should then be moved rapidly up and down, and then withdrawn in continuing rotary movements. Each application of the sponge must not last more than two or three seconds, but the instrument must be introduced again and again until the mucus and false membrane which are heard rattling in the trachea are entirely removed.

2. *Injection of tepid water.* "Before commencing the sponging, it is generally necessary to inject half a spoonful of warm or cold water into the trachea. This will facilitate the removal of the false membranes." The frequent introduction of a few drops of tepid water will often be found useful in diluting the mucus, and in rendering the expectoration of it much easier.

3. *Cauterization.* "This is performed in two ways; 1st, by touching; 2d, by instillation.

*Cauterization by touching.* "The sponge is imbibed with a caustic solution, and then applied directly upon every point of the mucous membrane which can be reached. This should be repeated at least three times the first day, and twice the two following days.

*Cauterization by instillation.* "The caustic solution is permitted to drop from a quill into the trachea, profiting as much as possible by a movement of inspiration; immediately after this water should be injected, and the sponge then vigorously re-applied until the air tubes are well cleaned. This species of cauterization should be repeated two or three times during three days." If, after the operation, it is discovered that there are no croupal concretions beyond the larynx, we must content ourselves with the cauterization by touching." For the first species of cauterization M. T. uses eighteen grains of nitrate of silver to the ounce of distilled water, for the second four grains only to the ounce. "The strong solution should never be instilled as it would occasion immediate coagulation of the mucus, and perhaps give rise to asphyxia." M. Bretonneau does not use cauterization by instillation, and M. T. himself cautions against their too frequent employment.

It will be necessary to mention in this place that cauterizations are not invariably recommended. M. T. has himself lately omitted them after his operations, and relied entirely upon sponging and the instillations of tepid water; in eight successive children treated in this manner three have been cured. This we shall have to consider as an unsettled point until future experience comes to prove to us which of the two methods is preferable.

Whenever the canula is removed for the purpose of cleaning it, or to permit us to sponge the trachea and bronchia, great care must be taken to introduce immediately the dilator, so as to maintain a free passage to the air. These various manuœvres should always be performed by the physician himself: this we would insist upon, for we have found several cases recorded where death has been occasioned from the impossibility on the part of the assistants of introducing the canula in time, which had been removed or had accidentally escaped from the trachea.

4. *Diet.* M. T. insists upon the necessity of giving good nourishment, such as milk, light soups, &c., during the traumatic fever, and as soon as the fever has ceased he recommends a still more nourishing diet; if there be but little appetite, and the strength of the patient does not improve, the wine of bark should be ordered.

We say nothing of the treatment which the peculiar state of the patient or a co-existing disease may require, for this of course must be directed upon general principles.

We shall not stop to answer at length the various objections which have been urged against tracheotomy; the success which has already been obtained is the best argument that can be produced to prove how unfonnded are most of them. As an operation tracheotomy is not dangerous: such is the opinion of the majority of the French surgeons, so far as we can learn, and this is confirmed by the happy result which we have seen follow the operation when performed for other diseases, no less than by the mass of facts which we find in the various periodicals. But it has been said, that pneumonia was of more frequent occurrence in croup after the operation, and such, to a certain extent, is the opinion of M. Trousseau, as he has himself informed us. To this we will answer, that thus far this point has not been proved; before this can be done it will be necessary to compare a large number of cases of equal severity, and under similar circumstances, upon half of which tracheotomy has been performed, and upon the other half not.

In the absence of such proof we are willing to grant that the assertion appears very plausible *a priori*, but if we examine a little into the facts, we shall be forced to admit that it is exceedingly unphilosophical to attribute entirely to a traumatic cause a disease which was already impending. Who is not aware of the frequent occurrence of pneumonia in the last stages of the diseases of children, in croup among others, especially when the patients have been much enfeebled and exhausted by the combined effect of a debilitating treatment and a harassing disease; added to this, we have in croup more or less congestion of the lungs, favoured by an imperfect äeration of the blood, impeding the pulmonary circulation, the dorsal decubitus, &c., &c. We have already all the elements necessary to the development of a pneumonia, and if the disease has not already commenced in many instances, is it not at least rational to suppose that nature is often so much exhausted, that even if air, which she so loudly demands be at last allowed her, she cannot rally sufficient force to drive back into the torrent of the circulation the mass of blood which operates like a foreign body in paralyzing her vital organs? These considerations induce us to believe that the irritation of the canula, of the cauterization, of the air itself, or the propagation of the inflammation from the wound, can rarely do more, in the majority of cases, than hasten a result which is often inevitable. Should future experience ever prove us to be wrong in our opinion in this respect, we shall still not see in the risk of producing a pneumonia an objection to tracheotomy. Ought one to hesitate in his choice who sees a child in the last period of croup, and on the one hand death morally certain, and on the other the danger of producing a pneumonia, but still a reasonable hope of saving life?

During our residence in Paris we have seen five children who were operated upon for croup, and the details of a sixth have been communicated to us; of these six four have been successful, viz.: two by M. Trousseau, one by M. Gerdy, and one by M. Nelaton. Two unsuccessful, viz.: one by M. Gerdy, and one of M. Trousseau's, upon whom the operation was performed by his interne, M. Sterling. We have already referred to the latter case, we shall now annex the four others.

*Case* 1. *Croup—Tracheotomy, performed by Professor* TROUSSEAU—*Cure.*

[For this case we are indebted to the politeness of M. Dupré, in whose practice it occurred.]

Jules ——, four years of age, fair hair, blue eyes; skin fine and white; temperament lymphatic. This child, born of parents who enjoy good health, is often indisposed; he has already had bronchitis and slight catarrhs, on several occasions. On the 1st of January, 1838, he had an attack of laryngite striduleuse, without precursory symptoms, but this false croup yielded in eight or ten days to the appropriate remedies.

On the 16th of October, 1839, M. Dupré was again consulted about this child. Under the influence of abundant rains, then very frequent, he had caught cold six days before; bleeding at the nose since three days; fever in the evenings; face pale; cough smothered; hoarseness; râle a a little sonorous on the right side posteriorly; redness of the fauces; the gaiety of the child has, however, but little diminished. About 5 o'clock he dined, and went to bed soon after.

Towards 11 o'clock at night, after a sleep slightly agitated by fever, he suddenly started out of his sleep; cough hoarse and violent during several minutes. M. Dupré arrived, and found the child drowsy, as if about to fall asleep; face very pale; respiration hurried, but little wheezing; voice hoarse; redness of the throat, without tumefaction of the tonsils; sonorous râle to the right posteriorly, but little to the left.

℞.—Syr. Ipecacuanha, ℥ss., Tart. Emet., gr. j.

The matter vomited, consisted of what the patient had eat at dinner, with a little tenacious mucus. (Sinapisms.)

*Oct.* 17*th.*—The night has been agitated; this morning the wheezing is more marked, and has become noisy, (bruyant;) hoarseness of the voice and of the cough; upon the left tonsil we remark three white patches, of the size of a grain of hempseed, and redness of the fauces; rhonchus same as before.

℞.—Five leeches to the front of the larynx; tonsil to be touched several times with powdered alum.

Considerable blood was lost, but there was no relief; at 12 o'clock another emetic was given, and the tonsils retouched with alum. The white patches have extended upon the pharynx, and reached the other tonsils. The matter vomited consists of tenacious mucus, without any false membrane.

In the afternoon mercurial frictions were ordered to the arm-pits and throat. Sinapisms.

*Evening.*—Remarkable paleness of the face; head thrown back; agitation; oppression; respiration high, tracheal; aphonia nearly complete; cough, with suffocation; pulse strong, 150 pulsations; false membranes cover the whole of the posterior part of the throat.

℞.—Cauterizations, with a solution of nitrate of silver, ʒj to an ounce of water, each time fragments of false membrane are brought away by the sponge.

18*th.*—Same state during the night; the cauterizations and the mercurial frictions have been continued. Respiratory murmur scarcely heard; tracheal râle more marked; paleness of the face; scarcely any engorgement of the cervical ganglions; perspirations from time to time. Suffocation appears so imminent that the operation of tracheotomy is deemed necessary. It was performed at half past one by Professor Trousseau.

As soon as the trachea was opened, portions of false membrane, mingled with blood, were ejected with force. A calm respiration soon succeeds, to the general state of agony which existed before the operation. In the evening, the pulse fell to 140, and the respiration was 32.

*Oct.* 19*th and* 20*th.*—The canula is changed every six hours, which is followed by a state of calm. The return of the wheezing and high respiration, the cough, with efforts to expectorate, the false membranes, indicate the necessity of cleaning the instrument. In the middle of the day the pulse is from 140 to 150.

℞.—Barley water with cherry syrup, a few grapes; injection.

21*st and* 22*d.*—The bronchiæ are more frequently obstructed by thick mucus; in spite of the frequent instillations of tepid water, the respiration becomes wheezing, and the cough suffocating, every two or three hours, or more; finally, after violent efforts of expulsion, a matter, like partially dried mucus, is thrown out with force.

Cauterization of the borders of the wound, which are covered with a grayish plastic membraniform exudation.

23*d.*—Same state; return of the suffocation nearly every hour; however, the prognosis is favourable, for during the absence of cough, a vesicular and puerile respiration is every where heard; the red, and almost rust-coloured mucus which is expectorated to-day, cannot, therefore, be a sign of pneumonia, but is probably owing to the accidental mingling of a little blood, coming, perhaps, from the rupture of some bronchial capillaries.

24*th.*—Last night has been the worst which the patient has passed since the operation.

It was remarked on this day that the mucus was expectorated by the mouth during the efforts of cough; at the same time we heard a slight laryngeal noise; the patient can articulate a few words in a low voice. The general state is better; pulse 134.

The canula is ordered not to be replaced. The opening in the trachea to be partially covered from time to time with a piece of English taffeta. The child suffers no inconvenience from this. Soup for diet.

From the 26th to the 30th, local and general amelioration progressive; abundant expectoration by the artificial opening, and by the mouth, of a whitish tenacious mucus; voice egophonic.

*Nov.* 7*th.*—The wound is completely cicatrized, but the patient continues to have a little fever in the evening, and agitation during sleep; cough with mucous râle on the right side, posteriorly, as before the commencement of the croup; the strength does not much improve, and there remains a pale and wan appearance of the skin.

The delay which the convalescence now experiences may be explained from the fact that the patient has left the spacious and well ventilated apartment where he remained during the activity of his disease, and has returned to the porter's lodge inhabited by his parents, where he is placed in the worst hygienic conditions,—deprived of air, sun, and light, sleeping in a small chamber, with his father and mother close by his side.

From the 7th to the 20th, strength does not improve; the child can scarcely sustain himself upon his legs; slight fever during the day and night; moisture of the hands; cough short, frequent, with oppression; mucous râle on the right side posteriorly; the appetite remains good. (Blister to the arm, then a second upon the chest laterally; gelatinous baths.) Scarcely any improvement; from the character and persistence of the symptoms, Dr. Dupré suspects the existence of miliary tubercles.

*Remarks.*—As we were present at the above operation, we may be permitted to make a few remarks to the interesting case of Dr. Dupré, of which we have given the translation. The symptoms of croup were here perfectly characterized, and their gravity left to us no hopes of a happy issue, except in an operation: in addition to the symptoms indicated, we will mention another, which alone would have been sufficient to enable us to prognosticate an approaching death; we refer to the extended movements of the sternum,—the lower extremity of which, at each inspiration, must have nearly touched the vertebra. The condition of the patient was such as we regard the most favourable for the operation; he had not been exhausted by a too great loss of blood, nor by the duration of the disease, and there was no reason to suspect either a pneumonia or a considerable engorgement of the lungs. The operation was performed very slowly, and with great care, by M. T. A large thyroid vein presented itself in the very track of the knife; this was carefully carried to one side by means of a blunt hook; a small artery sprung, but ceased giving out blood in two or three minutes; no ligatures were applied, and not an ounce of blood was lost. The moment the air found a free passage into the lungs, through the opening of the trachea, the respiration became perfectly calm, and the movements of the sternum and ribs became natural. No accident occurred; and in due time the canula was removed, and the child perfectly cured of its croup. If the convalescence become suddenly arrested, this should not be attributed to the operation, but to the more natural causes pointed out by M. Dupré.

For the following cases I am chiefly indebted to my friend, Dr. Trudeau: it will be necessary to add, that the circumstances under which the patients were observed, did not admit of more details.

*Case* 2. *Croup—Tracheotomy, by Professor* GERDY—*Cure.*

Louisa B., six years of age, slight of frame, and of a feeble constitution, entered the hospital St. Louis, in the service of M. Gerdy, the 11th of August, 1838. For several days this child had been affected with an acute bronchitis; but her disease presented nothing alarming until the night of the 9th, when she was awakened by a difficulty of respiration and an aggravation of her cough, which had become slightly metallic; a foot bath was administered by her mother. The day following the child was tranquil and gay; at the same time, the cough appeared less frequent, and of a less alarming character.

During the night of the 10th the paroxysms returned with greater energy than ever, and on the following day she was brought to the hospital.

The physician on duty visited the patient immediately after her admission, and found her in the following state:—Anxiety of countenance; frequent paroxysms of cough, which present a certain degree of hoarseness, with a slight metallic character; between the paroxysms the respiration was loud and wheezing; no expectoration. (Leeches to the throat; sinapisms.) After the leeches had fallen off, half a grain of tartar emetic was administered; during the vomiting produced several fragments of false membrane were thrown up.

After the emesis, the anxiety and oppression became diminished, and the pulse less frequent. The physician was called again during the night, in consequence of an aggravation of symptoms. Another emetic was administered, which, however, did not produce vomiting. The symptoms became more and more alarming.

*August* 12*th.*—Morning visit. Expression extremely anxious; face pale; lips blue; complete aphonia; considerable dyspnœa; respiration loud and wheezing; sibilant râle throughout the chest; pulse very frequent, cannot be counted.

The child appeared in a state of imminent suffocation, so that M. Gerdy immediately performed tracheotomy. The larynx and trachea were then carefully cleaned by the aid of the sponge of M. Bretonneau; they were then cauterized by a solution of nitrate of silver, gr. iv. to ℥j. of distilled water. The double canula of M. G. was afterwards introduced. The symptoms of asphyxia disappeared immediately after the operation; the respiration became comparatively easy; the pulse fell to 130.

13*th.*—Patient passed a good night; dyspnœa moderate; sibilant râle as before; pulse 130; considerable quantity of coagulated mucus passes by the canula. Cauterization; instillations of tepid water. (℞. Pectoral mixture; purgative injection; blister to chest.)

14*th.*—Decided improvement; the child is already gay; cough and respiration less frequent; expectoration of fragments of false membrane through the wound since yesterday. (Soup.)

15*th,* 16*th, and* 17*th.*—The improvement continues without accident.

18*th.*—The canula was withdrawn, and the opening closed with the finger; the child made signs to replace the canula. Respiration easy; a little mucus escapes through the canula, but no false membrane. (Cauterization to be continued.)

22*d.*—The canula is closed from time to time, in order to favour the respiration, which commences to pass by the glottis.

28*th.*—Canula withdrawn for two hours; without it, respiration still deficient.

31*st.*—Canula was permanently withdrawn, and a piece of dried lint introduced; aphonia persists. The wound was entirely cicatrized on the 10th of September; and on the 16th of the same month, the child left the hospital perfectly cured.

*Case* 3. *Croup—Tracheotomy, by* M. Nelaton, *Agrégé à la Faculté de Médecine de Paris—Cure.*

The daughter of Mr. ——, shoemaker, living near the hospital St. Louis, a child of fair complexion, well made, and of a good constitution, was remarked to be duller than usual, and refused to play on the 18th of January, 1839; no other symptoms attracted the attention of the parents. On the morning of the 19th, at 3 o'clock, A. M., she was seized with a cough so frequent, and of such an alarming character, that an "officier de santé" was immediately sent for. A pectoral infusion, and a sinapism to the neck, were ordered. At 10 A. M. of the same day, M. Nelaton was sent for. At this time, the child presented the following symptoms:—Aphonia very considerable; cough frequent, slightly stridulous; respiration oppressed; auscultation indicated a general sibilant râle. (℞. Tart. Emet. gr. ss., which was soon repeated.) Emesis was produced, during which a substance resembling false membrane, was thrown up; an abundant perspiration followed the emetic.

*Jan.* 20*th.*—Anxiety; but little cough; symptoms of imminent suffocation; stridulous respiration. Pulse frequent; skin hot. Tracheotomy was deemed necessary, and immediately performed by M. N. The only particularity in the operation was the quantity of blood that was lost, estimated at ℥xij. After the air had passed freely through the opening into the trachea, the hæmorrhage so far ceased that ligatures were found unnecessary. After the operation, the child remained in a state of feebleness, but the respiration was much less laborious.

Cauterization of the larynx and trachea, by means of a sponge dipped in a solution of potassa gr. iij, aq. distillat. ℥j, a tubular-form membrane about two inches in length, was withdrawn. (Antispasmodic potion; trachea to be sponged once every hour.) In the evening the child felt much better; slight fever.

21*st.*—Respiration less easy, fever somewhat increased; constipation.

℞.—Calomel. gr. iij.

*Evening.*—No stool; one glass of Seidlitz water.

22*d.*—Same state; one stool; expectoration of mucus considerable.

The child was taken to St. Louis, and placed in the service of M. Gerdy. The same treatment was followed. Respiration became daily more easy; the secretion from the bronchiæ became thicker and more abundant; cough less frequent.

30*th.*—The canula can be closed for several minutes.

*February* 7*th.*—Respiration much easier; the canula can be kept out for several hours.—On the 10th it was entirely removed during the day, but replaced at night. There still remains a good deal of cough and expectoration, especially during the night.

15*th.*—Canula entirely withdrawn, and on the 17th, the child returned to its parents. The wound was not yet healed; it was ordered to be dressed with dry lint.

*Remarks.*—It is important to notice in this case, the quantity of blood that was lost from the division of the thyroid veins, and the effect of the re-establishment of the respiration, in so far moderating it as to render the ligature of these vessels entirely unnecessary. The well known happy effect of nitrate of silver in limiting certain inflammations, and in altering the morbid action, by its peculiar influence, would induce us *a priori* to prefer it as a caustic in these cases, to the solution of potassa, employed by M. Nelaton, though the latter has the advantage of not coagulating the mucus.

*Case* 4. *Croup—Tracheotomy, by Professor* GERDY.—*Pneumonia—Death.*

The son of Mr. Klein, living at La Chapelle, four years of age, robust, of an apparently good constitution, and thus far a healthy child, had been a little indisposed for about ten days with a slight cold, which did not, however, prevent him from being as playful as usual. On the 19th of July, 1838, his cold increased rapidly in severity, so that on the morning of the 20th, a physician was called in. At this time the pulse and respiration were said to be frequent; the cough paroxysmal and aphonia imperfect. (Leeches were ordered to the throat; an emetic administered, which produced vomiting; diet.)

*July* 21*st*, morning.—M. Gerdy is called in consultation; the child is in a state bordering upon suffocation; anxiety of the countenance; agitation extreme; face pale; respiration 39 to the minute; wheezing; sibilant râle heard over the chest; expansion of the lungs incomplete; paroxysms of cough frequent; aphonia incomplete; pharynx red; no false membrane observed; pulse 140; skin hot.

Tracheotomy was judged necessary and immediately performed by M. G.; as soon as the thyroid veins were divided, an abundant hæmorrhage followed, but ceased as the respiration became established, by means of the opening in the trachea; no ligatures were applied. The efforts of cough which followed the operation, forced through the artificial opening fragments of false membrane, and a quantity of mucus. The sponge of M. Bretonneau, dipped in a solution of nitrate of silver, gr. x. to ℥j. of water, was introduced for the purpose of cauterizing the mucous membrane; on withdrawing this instrument, we found attached to it a portion of false membrane, of a tubular form, two inches in length. After this, the canula was replaced without much difficulty. Immediately the agitation of the patient became much less. Cauterization of the larynx to be repeated twice a day; sponging every half hour; one glass of Seidlitz water; solution of gum syrup for drink; large emollient cataplasm to the chest. A medical assistant remained constantly by the side of the patient.

In the evening the pulse was stronger; respiration easy, and fallen to 30: on removing the canula, in order to sponge the air tubes, another portion of false membrane solidly fixed was withdrawn.

22*d*.—Sensible amelioration; cough less frequent; heat of surface less.

Cauterization; sponging; injection; cataplasm.

*Evening*.—Face flushed; lips of wound tumefied; percussion sonorous; sibilant râle; a solution of nitrate of silver, fir. iij. aq. ℥j. was injected through the canula; the effect of this was the immediate coagulation of the mucus, which the patient expectorated with great difficulty; to avoid this inconvenience, a small quantity of tepid water was introduced after each injection of the caustic solution; the expectoration became easier.

23*d*.—The patient has passed an easy night, and slept well; the canula was once withdrawn to be cleaned. Edges of wound much tumefied; pulse feeble; respiration 40, a little obscure on the right side behind, with a slight dulness on percussion. To-day, at the return of the physician after a short absence, the child was found nearly aphyxiated, from the escape of the canula, which the parents were unable to replace; the wound was immediately dilated by the aid of a pair of forceps, and the instrument replaced.

24*th*.—State less satisfactory; pulse 135; respiration 42; expectoration of mucus very abundant.

℞.—Calomel, gr. v.—large blister to right side.

25*th*.—Cough much more frequent; the mucus from the bronchiæ has acquired such a degree of plasticity, that it is found necessary to clean the canula every quarter of an hour; percussion still more obscure on the right side posteriorly.

26*th*.—The condition of the patient becomes more alarming; pulse 145, very feeble; pupils much dilated.

Sinapisms to legs, large cataplasm over the epigastrium.

Death at 11, A. M. The parents would not consent to an autopsy being made.

The following conclusions may, we think, be drawn from what precedes.

1*st*.—That tracheotomy, generally recommended by the French physicians and surgeons, ought always to be resorted to in croup as a last resource, inasmuch as it has saved a little less than one patient out of four, even when performed at the last extremity, and often under the most unfavourable circumstances.

2*d*.—That double pneumonia and advanced phthisis are almost the only positive contraindications; that the extension of the disease to the bronchiæ, and great feebleness on the part of the patient from profuse bleeding, or other causes, are unfavourable conditions, but that cures may even be obtained in such cases.

3*d*.—That as too great a loss of blood is always to be regretted, and its effect in curing or even in shortening the duration of the disease, questionable, we should employ venesection and leeches with much caution, and trust more to local caustic applications, so generally recommended of late.

4*th*.—That the operation should be performed when the disease has reached its third stage, when death seems inevitable, and before the patient has become too much exhausted, the lungs congested, &c. &c.

5*th*.—That the operation should be performed very slowly and cautiously, so as to avoid dividing the vessels as much as possible.

6*th*.—That the canula we employ should be of large size, and in proportion to the physical development of the patient.

7*th*.—That the minutest attention should be paid to the after-treatment, as upon this materially depends the ultimate success.

8*th.*—That pneumonia, when it occurs, should rather be attributed to the state of the lungs of the patient at the moment of the operation, than to the immediate effect of the operation itself, and that were it otherwise, tracheotomy would still be insisted upon.

It is hardly necessary for us to add, in concluding this paper, that our remarks have reference solely to the true membranous croup, or diphtherite trachéale of M. Bretonneau; care must be taken, therefore, not to confound with it the laryngite striduleuse, (Bretonneau,) false croup, (Guersent,) or spasmodic croup of other authors, a disease of an entirely different nature, of a mild character, yielding, ordinarily, to a mild treatment, and often ceasing spontaneously.

